# A phase II study of biweekly dose-intensified oral capecitabine plus irinotecan (bXELIRI) for patients with advanced or metastatic gastric cancer

**DOI:** 10.1038/sj.bjc.6603752

**Published:** 2007-05-01

**Authors:** S C Oh, H Y Sur, H J Sung, I K Choi, S S Park, J H Seo, Y T Jeen, H J Chun, S W Shin, Y J Mok, J S Kim, Y H Kim

**Affiliations:** 1 Section of Hemato-Oncology, Department of Internal Medicine, Korea University College of Medicine, 126-1, Anam-Dong 5Ga, Sungbuk-Gu, Seoul 136-705, Korea; 2 Department of General Surgery, Korea University College of Medicine, 126-1, Anam-Dong 5Ga, Sungbuk-Gu, Seoul 136-705, Korea; 3 Section of Gastrointestinal Disease, Department of Internal Medicine, Korea University College of Medicine, 126-1, Anam-Dong 5Ga, Sungbuk-Gu, Seoul 136-705, Korea

**Keywords:** irinotecan, capecitabine, stomach neoplasms

## Abstract

Capecitabine, a prodrug of 5-FU, has been reported to generate maximal tumour activity at tumour sites and/or to improve drug tolerability as compared with 5-FU infusion, and it has also been demonstrated to act synergistically with irinotecan against some solid cancers. A previous study concluded that dose-intensified biweekly capecitabine seems to be more effective at increasing both response rate and progression-free survival time than conventional dose and schedule of capecitabine in colon cancer. We conducted this study to ascertain the efficacy and toxicity of dose-intensified biweekly capecitabine and irinotecan combination chemotherapy in chemotherapy-naïve advanced or metastatic gastric cancer patients. Patients were treated with irinotecan 130 mg m^−2^ intravenously for 90 min on days 1 and 15. Capecitabine at 3500 mg m^−2^ day^−1^, divided into two sessions per day, was administered for seven consecutive days from days 1 and 15, and followed by a 7-day drug-free period, respectively. Fifty-five eligible patients were enrolled in this study from November 2003 to April 2006. There were 22 women and 33 men: median patient age was 54 years (range: 27–81). A total of 200 treatment cycles were administered at a median number of four per patient (range: 1–9). Intent-to-treatment analysis showed that one patient achieved complete response (1.8%), 23 partial response (41.8%), 15 stable disease (27.3%), 10 progressive disease (18.2%) and 6 were non-evaluable (10.9%). The overall response rate was 43.6% (95% confidence interval: 30.2–56.9). The common grade 3–4 toxicities were neutropenia in 12 (21.8%), nausea/vomiting in 3 (5.4%) and diarrhea in 4 (7.2%) patients. Median time to progression was 5 months (range: 0.5–11 months), median survival duration was 11 months (range: 0.5–45 months) and median response duration was 6 months (range: 0.5–9 months). Biweekly dose-intensified capecitabine and irinotecan combination chemotherapy was active for the treatment of advanced or metastatic gastric cancers with a tolerable safety profile.

Gastric cancer is the second most common cancer and one of the most common causes of cancer mortality in the world (Boring *et al*, 1994; Shin *et al*, 2005; Kamangar *et al*, 2006). Despite reducing gastric cancer incidence in the Occident, the incidence of cardiac and gastroesophageal junctional adenocarcinoma has increased, and the 5-year survival rate for advanced gastric cancer has not significantly changed (Brown and Devesa, 2002). The only curative treatment modality for gastric cancer is surgery. However, many patients present with advanced stage disease, and the relapse rate of those who are subjects for curative resection is high, at around 65% (Hartgrink *et al*, 2004). Thus, most gastric cancer patients are subjects for systemic chemotherapy. Current response rates to chemotherapy among advanced gastric cancer patients are between 10 and 20% for a single agent and between 30 and 50% for combination chemotherapy (Barone *et al*, 1998; Ajani *et al*, 2005; Moehler *et al*, 2005; Ohtsu *et al*, 2006; Nardi *et al*, 2007). However, during recent years, several new agents, for example, taxane, irinotecan and oxaliplatin, have been introduced for the treatment of gastric cancer (Louvet *et al*, 2002; Pozzo *et al*, 2004; Roth *et al*, 2004), and these drugs have shown promising response rates without sacrificing acceptable toxicity.

Irinotecan (Camptor®; Pfizer) is a semisynthetic plant alkaloid camptothecin, which inhibits DNA topoisomerase-I. SN-38 has been identified to be the important metabolite of irinotecan, and to inhibit the regulation of DNA during cell replication. In recent meta-analysis of chemotherapies used to treat advanced gastric cancer, a comparison between irinotecan-containing versus nonirinotecan-containing combinations (mainly 5-FU/cisplatin) showed a non-survival benefit in favour of irinotecan-containing regimens (HR=0.88) (Wagner *et al*, 2006).

Capecitabine (Xeloda®; Roche) is an oral fluoropyrimidine that selectively generates higher levels of 5-FU in cancer tissues than in normal tissues via the action of thymidine phosphorylase. Capecitabine monotherapy was proved to be active against advanced gastric cancer with the response rate of from 19.4 to 34% (Koizumi *et al*, 2003; Hong *et al*, 2004); moreover, median survival duration in these studies was comparable to other double or triple combination chemotherapies. Capecitabine has the advantages of convenient oral administration and of mimicking the effect of protracted intravenous (i.v.) 5-FU.

Irinotecan and capecitabine in combination could be possible to have a synergistic effect in previous pre-clinical and clinical studies (Guichard *et al*, 1998; Mullany *et al*, 1998; Azrak *et al*, 2004; Cao *et al*, 2005; Rea *et al*, 2005). Irinotecan reduces DNA synthesis, increases dTTP pools and inhibits dUMP synthesis, which are also associated with the antitumour activity of capecitabine (Mullany *et al*, 1998). Actually, irinotecan/capecitabine combination regimens have been used to treat several types of solid tumours (Borner *et al*, 2005; Han *et al*, 2005; Kim *et al*, 2005; Baek *et al*, 2006).

However, no optimum schedule has been identified for these two drugs. This is the first trial of high-dose regimen compared to usual capecitabine schedules and it is biweekly rather than triweekly against advanced gastric cancer. A preclinical study using human tumour xenografts indicated that tumour growth inhibition depends on the total dose of capecitabine administered and not on the dosing schedule (Van Cutsem *et al*, 2000), because short-term intermittent rather than prolonged or continuous administration may allow capecitabine dose intensities to be increased (and thus antitumour efficacies) without increasing toxicity via the incorporation of longer drug-free intervals. Actually, this dose-intensified biweekly administration seems to be more effective at increasing response rate and progression-free survival than conventional dose and schedule of capecitabine in colon cancer, without compromising its safety profile, despite the fact that capecitabine was administered with oxaliplatin (Thomas *et al*, 2001; Scheithauer *et al*, 2002, 2003). In addition, favourable results were reported for higher cumulative doses of oral capecitabine in advanced gastroesophageal cancer, although the administration schedule used differed from that used in the present study (Van Cutsem *et al*, 2000).

Here, we report the findings of a phase II study that was undertaken to evaluate the efficacy and toxicity of biweekly irinotecan and high-dose capecitabine combination chemotherapy for the treatment of advanced and/or metastatic gastric cancer in a first-line treatment setting.

## PATIENTS AND METHODS

Between November 2003 and January 2006, 55 patients were enrolled in this trial. Written informed consent was obtained from all study subjects, and the study protocol was approved by our institutional review board. All patients had histologically proven gastric adenocarcinoma and locally unresectable or recurrent/metastatic disease. Only patients with measurable disease were accepted. The eligibility requirements included a performance status of ⩽2 and a life expectancy of at least 12 weeks. Preclinical laboratory parameters included an absolute neutrophil count of >1.5 × 10^3^ l^−1^ and a platelet count of >100 × 10^3^/ l^−1^. Patients were required to have normal cardiac, renal and hepatic functions. Contraindications to entry included any active infectious process, active heart disease or any concomitant second primary cancer. Patients who had undergone previous chemotherapy, except adjuvant chemotherapy completed within 6 months before enrollment, were also excluded.

### Treatment

Irinotecan 130 mg m^−2^ was administrated i.v. for 90 min on days 1 and 15. Capecitabine 3500 mg m^−2^ day^−1^ b.i.d. was administered for seven consecutive days from days 1 and 15, which was followed by a 7-day drug-free interval, respectively.

If a patient developed neutropenia (an absolute neutrophil count of <1000) of >5 days duration or febrile neutropenia, irinotecan and capecitabine dosages were reduced by 25% during subsequent treatment cycles. Treatment was interrupted when a patient had a non-haematologic toxicity of >grade 2 during treatment, and restarted at a 25% reduced dosage after the toxicity had resolved to a level of <grade 2. If a patient had severe toxicity at the time of a scheduled retreatment, drug administration was postponed until the toxicity had resolved. If drug administration was delayed for more than 3 weeks, the patient was excluded from the study. When a patient had diarrhea, oral loperamide was immediately started at 2 mg, and subsequently 1 mg was given every 2 h until symptoms were relieved. Acute onset diarrhea that occurred within 24 h was considered as a cholinergic symptom and was prevented with atropine. This above cycle was repeated every 4 weeks until tumour progression or the development of treatment intolerance.

### Evaluation

All patients were examined clinically before enrolment by laboratory evaluation, which included a complete blood cell count and electrolytes, blood urea nitrogen, creatinine, calcium, phosphorous, bilirubin, alkaline phosphatase, aspartate aminotransferase, alanine aminotransferase, total proteins and albumin. Upper gastrointestinal endoscopy, abdominal-computed tomography and other appropriate procedures were also performed. Complete blood cell counts were examined on days 1 and 14 of every cycle, and chest radiography, liver function tests, blood urea nitrogen, creatinine and electrolyte were examined on day 28 of every cycle. Examination of the evaluable parameters such as abdominal-computed tomography for evaluating of response was done after every two cycles of treatment. Response evaluation criteria in solid tumours were adopted to evaluate treatment response (Therasse *et al*, 2000). Toxicity was reported using a World Health Organization (WHO) Toxicity Criteria

### Statistical analysis

Response rate was used as the primary end point in the present study, and a response rate of 40% was anticipated. A response rate of 20% was considered to be the minimum level for continuing the study. The *α*-level of the design used was 0.05 and its power was 0.9. The number of subjects was calculated using the Simon two-stage optimal Minimax design (Simon, 1989). A total of 45 evaluable patients were needed; 24 patients were planned for stage 1 and 21 for stage 2. If five or fewer responses had been observed during stage 1, then the trial would have been stopped. The proportion of patients who responded to treatment was used to estimate the true response rate with a 95% confidence interval (CI). Actuarial survival duration was determined using the Kaplan–Meier method. Response duration was calculated from the date of response confirmation to the date when disease progression was first observed. Survival duration was calculated from the first day of treatment until death or last follow-up. Time to disease progression was calculated from the first day of treatment to the date when disease progression was first observed.

## RESULTS

### Patient's characteristics

Fifty-five patients were enrolled in this trial. Patient characteristics are listed in Table 1. All patients were evaluable for toxicity, but only 49 patients (after excluding three patients who withdrew consent and three patients who failed to continue treatment due to adverse events after one cycle of chemotherapy) were evaluable. Median patient age was 54 years, and ranged from 27 to 81 years. Thirty-three patients were male and 22 were female. All patients had a good performance status of <grade 2. Twelve patients had a history of surgery (with curative intent for eight patients and with palliative intent for four), and only four patients had received adjuvant chemotherapy. Forty-three patients were treatment näive.

### Response to chemotherapy

Fifty-five enrolled patients received 200 chemotherapy cycles; a median number of four per patient, which ranged from one to nine (Table 2). Six patients were non-evaluable; three patients refused further treatment after one cycle of chemotherapy and asked to be referred to other hospital, and treatment was discontinued in three patients due to toxicity after one cycle of chemotherapy. However, all six were included in the toxicity evaluation and in response rate calculations. By intention-to-treat analysis, one of the 55 patients achieved complete response (1.8%) and 23 showed partial response (41.8%). The overall objective response rate was 43.6% (95% CI: 30.2–56.9%) with a median response duration of 6 months (range: 0.5–9 months). Fifteen patients (27.3%) had stable disease and 10 patients (18.2%) had disease progression as a best response.

Median time to progression was 5 months (range: 0.5–11 months), and median survival duration was 11 months (range: 0.5–45 months).

### Toxicity

Fifty-five patients were evaluable for toxicities (Table 3). The most commonly encountered haematologic grade 3 or 4 toxicity on irinotecan/capecitabine was neutropenia. Grades 3 and 4 neutropenia were experienced by eight (14.5%) and four patients (7.3%), respectively. Grades 3 and 4 anemia were also observed in five (9.1%) and one patient (1.8%), respectively. The most common non-haematologic toxicity was diarrhea. Grades 3 and 4 diarrhea both occurred in two patients (3.6%).

Fourteen patients required irinotecan and capecitabine dose modifications. Of these, the most common causes of dose reduction were prolonged grade 3 or 4 neutropenia in five patients. Other causes of dose reduction were grade 3 or 4 nausea/vomiting in one patient, grade 4 diarrhea in one patient and grade 3 fatigue in one patient. Another five patients had a capecitabine dose modification. The most common causes of these dose reductions were grade 2 or 3 hand/foot syndrome in three patients. Another two patients underwent capecitabine dose reduction due to investigator discretion for grade 3 fatigue in one patient and grade 3 hepatitis in the other. Three patients discontinued treatment due to toxicity after one cycle of chemotherapy; grade 3 ischemic colitis in one, bowel perforation in one and death from pneumonia in one. In total nine (4.5%) cycles were delayed. The most common cause of treatment delay was neutropenia in four patients; other causes were nausea/vomiting in three patients, fatigue in one and fever in one.

**Figure 1:**
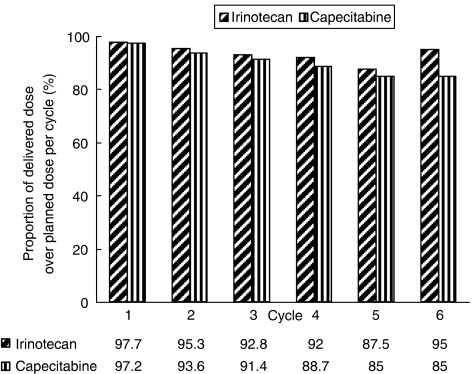
Proportion of delivered dose over planned dose per cycle and per patient during respective treatment cycles.

The mean dose intensities of irinotecan and capecitabine were 61.5 mg m^−2^ week^−1^ and 11 343 mg m^−2^ week^−1^, respectively, which were 94.7 and 92.6% of their respective intended doses (Figure 1).

## DISCUSSION

Pozzo *et al* (2004) compared irinotecan/cisplatin and irinotecan/5-FU/leucovorin with the aim of selecting a better regimen for a comparative phase III study with cisplatin/5-FU for the treatment of advanced gastric cancer, and the overall response rate of the irinotecan/5-FU/leucovorin arm (42%) was found to be superior to that of the irinotecan/cisplatin arm (32%). In the present study, we used capecitabine instead of 5-FU and leucovorin. Capecitabine is an oral fluoropyrimidine, and selectively produces higher concentrations of 5-FU in tumour tissues than in normal tissues. Hong *et al* (2004) studied capecitabine monotherapy for the treatment of advanced gastric cancer, and used capecitabine at 1250 mg m^−2^ twice daily (2500 mg m^−2^ day^−1^) for 14 days followed by 7 days of rest, for up to six cycles. Rates of overall response and stable disease were 34 and 30%, respectively, median time to progression was 3.2 months and median overall survival was 9.5 months. The most common toxicity was hand–foot syndrome, but grade 3 or 4 hand–foot syndrome was detected in only 9% of patients. Different capecitabine schedules have been examined for the treatment of advanced gastric cancer (Koizumi *et al*, 2003). In this earlier study, capecitabine as a single agent was administered at 828 mg m^−2^ b.i.d. for 3 weeks, followed by 1 week off, and a response rate of 19% (95% CI: 7.5–37.5%) was observed in gastric cancer. Median time to progression was 85 days and median survival was 247.5 days. No toxicity of ⩾grade 3 was reported, and the toxicity of capecitabine appeared to be correlated with the drug administration schedule.

The aim of this study was to determine the response rate and toxicities of combined dose-intensified biweekly capecitabine and irinotecan for the treatment of advanced gastric cancer. Capecitabine in the combination chemotherapy was reported to be at least as effective as 5-FU and leucovorin without additional toxicity in a REAL-2 study (Sumpter *et al*, 2005). Moreover, capecitabine has several advantages over i.v. 5-FU for clinical applications – the first of which is convenience. Capecitabine can be administered enterally without a portable infusion pump and central venous line, which results in high drug compliance and the maintenance of a constant 5-FU plasma level. The second reason concerns its safety, that is, capecitabine can be discontinued whenever severe toxicity develops, which prevents toxicity-induced deterioration. Our results demonstrate that dose-intensified biweekly capecitabine and irinotecan are active against advanced gastric cancer with a 43% overall response rate (95% CI: 30.2–56.9%) and a median response duration of 6 months. Moreover, the results achieved in this study could be favourably comparable with those of other studies that used an irinotecan-based combination regimen (Pozzo *et al*, 2004; Baek *et al*, 2006). Biweekly irinotecan and capecitabine (180 mg m^−2^, day 1 and 2000 mg m^−2^, days 1–9, respectively) have also been studied against gastroesophageal cancer (Burge *et al*, 2006), and an overall response rate of 32% was obtained. Because previous studies have produced improved results for dose-intensified biweekly capecitabine against colorectal cancer, in the present study, we adopted this dose and schedule for capecitabine instead of the conventional triweekly regimen against gastric cancer. The dose-intensified biweekly capecitabine arm showed a higher response rate (54.2 *vs* 42.2%) and a longer median progression-free survival time than the low-dose arm (10.5 *vs* 6.0 months) without an increase in toxicities (Van Cutsem *et al*, 2000; Scheithauer *et al*, 2003). In a study by Scheithauer *et al*, colorectal cancer patients received oxaliplatin 130 mg m^−2^ on day 1 plus capecitabine 2000 mg m^−2^ day^−1^ on days 1–14 every 3 weeks or oxaliplatin 85 mg m^−2^ on days 1 and 15 combined with capecitabine 3500 mg m^−2^ on days 1–7 and 15–21 every 4 weeks. However, no difference was found between the rates of haematologic and non-haematologic toxicities for the biweekly and triweekly regimens. Moreover, despite the use of a greater dose intensity of capecitabine, toxicities were tolerable for most patients, which were contrary to expectation (Kohne *et al*, 2005).

Clinical trials with novel drugs introduced to treat advanced gastric cancer have prolonged survival time. In particular, taxane- or oxaliplatin-based combination chemotherapies have shown high response rates from 38 to 54% and a median overall survival time of from 8.6 to 11.4 months against advanced gastric cancer (Louvet *et al*, 2002; De Vita *et al*, 2005; Lordick *et al*, 2005; Van Cutsem *et al*, 2006). Median survival duration in the present study was 11 months, which is the highest recorded to date by gastric cancer studies.

In the present study, neutropenia was the most common grade 3 or 4 haematologic toxicity, and was demonstrated in eight (14.5%) and four (7.3%) patients, respectively. However, all these patients recovered with supportive care based on, for example, the administration of granulocyte colony stimulating factor.

As was expected, in the present study, the most common non-haematologic toxicity was diarrhea, which occurred in 19 patients (34.5%). However, grade 3 or 4 diarrhea occurred in only two patients (3.6%) each. Moreover, this 7.2% rate of grade 3 or 4 diarrhea was less than that observed in other studies, which reported rates of 15–22% (Kim *et al*, 2005; Baek *et al*, 2006; Burge *et al*, 2006). The diarrhea and hand–foot syndrome were dose-limiting toxicities in phase I studies using capecitabine (Budman *et al*, 1998; Mackean *et al*, 1998). In the present study, nine patients (16.4%) experienced any grade of hand–foot syndrome and only three patients (5.5%) experienced grade 3 hand–foot syndrome. The rate of hand–foot syndrome in the present study was definitely lower than those of triweekly capecitabine studies, which have reported rates between 40 and 71% (Park *et al*, 2004; Baek *et al*, 2006). Despite a high mean dose intensity, the rates of diarrhea and hand–foot syndrome in the present study were lower than those of triweekly capecitabine studies (Hong *et al*, 2004; Park *et al*, 2004; Han *et al*, 2005; Baek *et al*, 2006). The capecitabine dose intensity used in the present study was 30% higher than that used by Baek *et al* (i.e., 11 343 mg m^−2^ week^−1^ in our study *vs* 8641 mg m^−2^ week^−1^ by Baek *et al*) and dose intensity was maintained during continued treatment cycles. In addition, the dose reduction rate in the present study (38.7%) was lower than that found by Baek *et al* study (57%) (Baek *et al*, 2006). Given higher dose intensities and uninterrupted treatment due to toxicity development, biweekly dose-intensified capecitabine and irinotecan combination chemotherapy appears to offer the possibility of longer response and survival duration.

In conclusion, dose-intensified biweekly capecitabine and irinotecan combination was found to be a moderately active regimen against advanced gastric cancer with manageable toxicity. The convenience of administering this regimen and the low rate of non-haematologic toxicities encountered suggest that it should be considered an option for the treatment of advanced gastric cancer. Phase III-randomized clinical trials are warranted to compare the efficacies and safety profiles of biweekly dose-intensified administration and conventional triweekly capecitabine.

## Figures and Tables

**Table 1:** Patient characteristics

Total no. of patients	55
No. of evaluable patients	49
Median age in year	54
Range	25–81
Male/Female	33/22
	
*ECOG performance status*	
0	3
1	41
2	11
	
*Previous treatment*	
None	43
Operation	12
Palliative/curative	4/8
Chemotherapy	4
IP/adjuvant	2/2

**Table 2:** Treatment response in gastric cancer patients

Total no. chemotherapy cycles	200
Median	4
Range	1–9
	
*Dose delivery*	
Irinotecan (%)	94.7
Capecitabine (%)	92.6
Enrolled patients	55
	
* Response (%)*	
CR	1 (1.8)
PR	23 (41.8)
SD	15 (27.3)
PD	10 (18.2)
NE	6 (10.9)
Overall response (95% CI)	43.6% (30.2–56.9%)
Median time to progression, month (range)	5 (0.5–11)
Median survival duration, month (range)	11 (0.5–45)
Median response duration, month (range)	6 (0.5–9)

**Table 3:** Major toxicities – WHO criteria (*N*=55)

	**Number of patients (%)**		
**Toxicity**	**Grade 1/2**	**3**	**4**
Neutropenia	13 (23.6)	8 (14.5)	4 (7.3)
Thrombocytopenia	2 (3.6)	1 (1.8)	1 (1.8)
Anemia	16 (29.1)	5 (9.1)	1 (1.8)
Mucositis	3 (5.4)	0	2 (3.6)
Diarrhea	15 (27.3)	2 (3.6)	2 (3.6)
Nausea/vomiting	15 (27.3)	2 (3.6)	1 (1.8)
Hepatotoxicity	2 (3.6)	2 (3.6)	0
Hand–foot syndrome	6 (10.9)	3(5.5)	—
Alopecia	14 (25.5)	3 (5.5)	0
Fever	6 (10.9)	0	0
Nephropathy	1 (3.8)	0	0
Neuropathy	2 (3.6)	0	0
Fatigue	1 (1.9)	2 (3.6)	1 (1.8)
Colitis	0	2 (3.6)	0
Constipation	2 (3.6)	0	0
Infection	0	2 (3.6)	0
